# The complete mitochondrial genome of the *Capsaloides cristatus* (Platyhelminthes, Monogenea), a pathogen of the sailfish (*Istiophorus platypterus*)

**DOI:** 10.1080/23802359.2021.1899077

**Published:** 2021-03-18

**Authors:** Changping Yang, Yan Liu, Binbin Shan, Gongjun Zhang, Yu Zhao, Dianrong Sun, Wei Yu

**Affiliations:** a South China Sea Fisheries Research Institute, Chinese Academy of Fisheries Sciences, Guangzhou, China; b Key Laboratory of South China Sea Fishery Resources Exploration & Utilization, Ministry of Agriculture and Rural Affairs, Guangzhou, China; c Key Laboratory of Fishery Ecology and Environment, Guangdong Province, Guangzhou, China; d College of Aquatic products, Tianjin Agriculture University, Tianjin, China; e Shenzhen Base of South China Sea Fisheries Research Institute, Chinese Academy of Fishery Sciences, Shenzhen, China

**Keywords:** Mitochondrial genome, Monogenean, *Capsaloides cristatus*, sailfish, South China Sea

## Abstract

This study reports the complete mitochondrial genome of the *Capsaloides cristatus* (Monogenea: Capsalidae) collected from the gill lamella of *Istiophorus platypterus*. The total length of the mitogenome was 13,948 bp, containing 12 typical platyhelminthic protein-coding genes, 22 tRNA genes, 2 rRNA genes and a putative non-coding region, with the *atp8* gene being absent. The total A + T content was 65.99%, which was significantly higher than that of the C + G content (34.01%). There were two kinds of start codons (ATG and GTG) and three kinds of terminated codons (TAA, TAG and TGA) in the 12 protein-coding genes. Phylogentic analysis revealed close relationships among the genera *Capsaloides*, *Capsala*, *Benedenia* and *Neobenedenia* with high bootstrap value supported. This study will provide useful molecular data for a better understanding of the species identification and phylogenetic position of *C. cristatus*.

The family Capsalidae Baird, 1853 comprises approximately 200 monogenean species, most of which are ectoparasites on marine fishes and some are important pathogens of cultivated fishes. The taxonomic identities of the members of the genus *Capsaloides* Price, 1938 are not easy to confirm due to deficient descriptions or illustrations (or both) of many of the nominal species (Chisholm & Whittington, 2006). *Capsaloides cristatus* Yamaguti, 1968 is a common external pathogen of the sailfish and sometimes lead to inflammation, mucus hyperproduction and hemorrhage of their hosts (Paperna, 1991). According to Chisholm & Whittington (2006), *C. cristatus* can be distinguished with other species of the closely related species by the morphology of the haptoral accessory sclerites and the number and shape of dorsomarginal body sclerites. Though the general morphology is conserved, some morphological characters of *C. cristatus* can be easily influenced by various factors such as the parasitizing sites, the salinity and temperature in the environment, and the growth stage of parasite (Whittington et al., 2004). As a result, this study sequenced the complete mitochondrial genome of *C. cristatus*, aiming to provide useful molecular data for the identification and an improved understanding of the phylogenetic position of this parasite.

Specimens of *C. cristatus* were collected from the gill lamella of the Atlantic sailfish (*Istiophorus platypterus*) from Shanwei city, Guangdong Province, China (21°24′7.2″N, 115°19′51.6″E) in April 2019. The specimen was deposited in the Key Laboratory of South China Sea Fishery Resources Exploration & Utilization, Ministry of Agriculture and Rural Affairs, South China Sea Fisheries Research Institute, Chinese Academy of Fisheries Sciences under the voucher number CC20190912. By using the E.Z.N.A^®^ Tissue DNA kit (OMEGA, USA), the total genomic DNA was extracted from the tissue of *C. cristatus*. The paired-end DNA library with insert size of 300−500 bp was constructed and sequenced by next generation sequencing (Illumina HisSeq 4000). The clean data without sequencing adapters were *de novo* assembled by NOVOplasty software (Loh et al., 2016). The assembled mitochondrial genes were identified by BLAST searches in the NCBI database. The locations of the protein-coding genes (PCGs) were determined using ORF Finder via NCBI, and the tRNA genes were verified using the MITOS WebServer (http://mitos2.bioinf.uni-leipzig.de/index.py). The start codons and stop codons of PCGs were identified based on comparisons with other monogeneans. Within the range of the previously reported monogenean mitogenomes, the whole mitochondrial genome of *C. cristatus* was 13,948 bp in length. The total A + T content (65.99%) was higher than the C + G content (34.01%) with a low G content (19.06), indicating an anti-guanine bias of the mitogenome. The mitogenome of *C. cristatus* consisted of 12 typical platyhelminthic PCGs, 22 tRNA genes, 2 rRNA genes and a putative non-coding region. According with the previously reported monogeneans (Plaisance et al., 2007; Huyse et al., 2008; Perkins et al., 2010; Kang et al., 2012; Zhang et al., 2014; Yang et al., 2020), all of the mitochondrial genes of *C. cristatus* were transcribed from the heavy strand, with the *atp8* gene being absent.

All of the 12 PCGs found in other monogeneans were also present in *C. cristatus*, including one cytochrome b subunit (*cytb*), one ATP synthase subunit (*atp6*), three cytochrome c oxidase subunits (*cox1*-*3*), and seven NADH dehydrogenase subunits (*nad1*-*6*, *nad4L*). Three of the 12 protein-coding genes (*nad1*, *nad2* and *nad4L*) started with the codon GTG, while the left nine genes (*cytb*, *atp6*, *cox1*-*3* and *nad3-6*) used the start codon ATG. Interestingly, six of the 12 protein-coding genes (*cox1*, *cytb*, *nad1*, *nad3*, *nadL* and *nad6*) were inferred to end with the TAG terminated codon, four genes ended with the TAA terminated codon (*nad2*, *nad4, nad5* and *atp6*), while the gene *cox2* and *cox3* ended with the codon TGA and TA-, respectively. Among the protein-coding genes, the longest one was *cox1* with a length of 1572 bp, whereas the shortest one was *nad4L* (246 bp). The 22 tRNA genes folding into cloverleaf secondary structures were determined, with their sizes ranging from 58 bp to 71 bp. Separated by the *trnC* gene, the two rRNA genes *rrnL* and *rrnS* were located between *cox2* and *trnT*, with a length of 948 bp and 736 bp, respectively. In addition, the none-coding region was confirmed to be 735 bp in length, and was determined to locate between the *trnV* and *trnQ* gene. The A + T content (79.05%) of the non-coding region was significantly higher than that of the overall mitochondrial genome (65.99%). This result was similar to those previously reported by Kang et al. (2012) for *Benedenia hoshinai*, Zhang et al. (2014) for *Neobenedenia melleni* and Yang et al. (2020) for *Capsala pricei*.

To analyze the phylogenetic position of *C. cristatus*, we constructed a phylogenetic tree using the maximum likelihood (1000 bootstrap replicates) method on the basis of 12 PCGs of 17 monogenean species. For contrasting the tree topology, *Taenia solium* (Cestoda) was selected as outgroup (Yang et al., 2020). The phylogenetic tree showed that *C. cristatus* was clustered with *C. pricei*, *B. seriolae*, *B. hoshinai* and *N. melleni* (Figure 1), suggesting close relationships among the genera *Capsaloides*, *Capsala*, *Benedenia* and *Neobenedenia*. The complete mitochondrial genome sequence of *C. cristatus* provided important dataset for a better understanding of the species identification and mitogenomic evolution of monogeneans.

**Figure 1.**
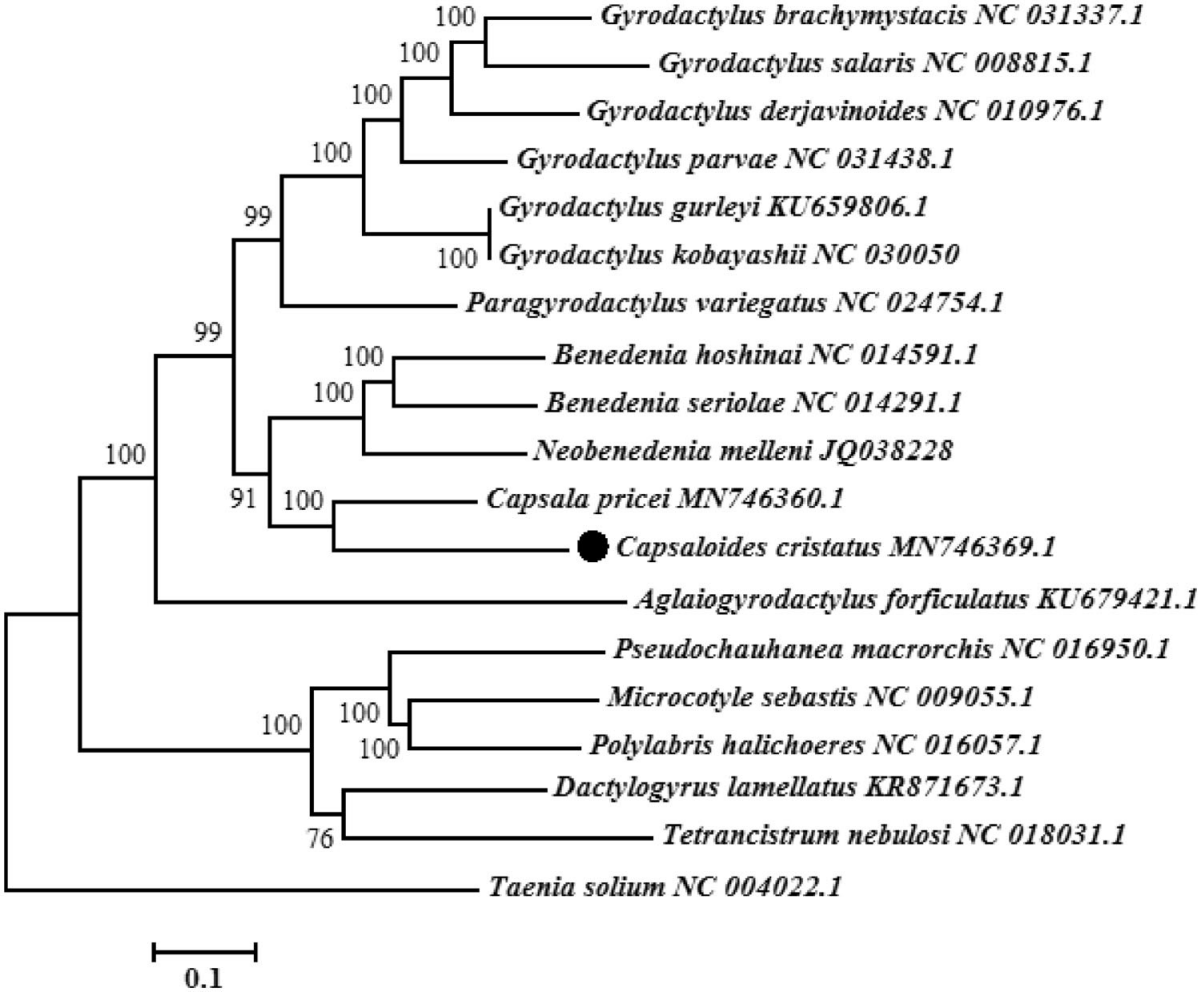
Phylogenetic tree of *Capsaloides cristatus* and other 18 flatworms (17 monogenean species and 1 outgroup species) based on 12 protein-coding genes.

## Data Availability

The genome sequence data that support the findings of this study are openly available in GenBank of NCBI at (https://www.ncbi.nlm.nih.gov/) under the accession no. MN746369.1. The associated BioProject, SRA, and Bio-Sample numbers are PRJNA592303, SRS5732812, and SAMN13420506, respectively.
